# The role of kidney biopsy in the diagnosis of membranous nephropathy

**DOI:** 10.1093/ckj/sfae292

**Published:** 2024-10-03

**Authors:** Dario Roccatello, Roberta Fenoglio, Savino Sciascia

**Affiliations:** University Center of Excellence on Nephrologic, Rheumatologic and Rare Diseases (ERK-net, ERN-Reconnect and RITA-ERN Member), Nephrology and Dialysis Unit and Center of Immuno-Rheumatology and Rare Diseases (CMID), Coordinating Center of the Interregional Network for Rare Diseases of Piedmont and Aosta Valley San Giovanni Bosco Hub Hospital, and Department of Clinical and Biological Sciences of the University of Turin, Turin, Italy; University Center of Excellence on Nephrologic, Rheumatologic and Rare Diseases (ERK-net, ERN-Reconnect and RITA-ERN Member), Nephrology and Dialysis Unit and Center of Immuno-Rheumatology and Rare Diseases (CMID), Coordinating Center of the Interregional Network for Rare Diseases of Piedmont and Aosta Valley San Giovanni Bosco Hub Hospital, and Department of Clinical and Biological Sciences of the University of Turin, Turin, Italy; University Center of Excellence on Nephrologic, Rheumatologic and Rare Diseases (ERK-net, ERN-Reconnect and RITA-ERN Member), Nephrology and Dialysis Unit and Center of Immuno-Rheumatology and Rare Diseases (CMID), Coordinating Center of the Interregional Network for Rare Diseases of Piedmont and Aosta Valley San Giovanni Bosco Hub Hospital, and Department of Clinical and Biological Sciences of the University of Turin, Turin, Italy

**Keywords:** membranous nephropathy, PLA2R antibodies, renal biopsy, Rituximab, serological diagnosis

## Abstract

The discovery of the target antigen M-type phospholipase A2 receptor (PLA2R) with the possibility to detect anti-PLA2R antibodies in serum as well as the identification of several other antigens, overall accounting for almost all cases of membranous nephropathy, paved the way to a revolutionary change in the classification of membranous nephropathy. Serum anti-PLA2R autoantibody titers have been found to be highly specific diagnostic and prognostic biomarkers. Therefore, a positive test for anti-PLA2R serology in patients who present with nephrotic syndrome, normal kidney function, and no evidence of another process to account for proteinuria is believed to suffice to make a diagnosis of primary membranous nephropathy, thus removing the need for a renal biopsy. While technological advances will likely allow this proposal to prevail in the near future, the reasons why renal biopsy could still remain a critical tool for the management of membranous nephropathy in real life are discussed.

## CLINICAL CASE

A 43-year-old woman underwent nephrological observation due to a 6-month history of nephrotic syndrome (proteinuria 7 g/day, serum albumin 2.6 g/dl) with normal kidney function [serum creatinine (sCr) 1.1 mg/dl, glomerular filtration rate 96 ml/min], peripheral edema, and normal blood pressure. Further examinations, including an extensive evaluation of autoimmunity (Supplementary Table S1), were normal, except for phospholipase A2 receptor (PLA2R) antibodies, which tested positive at high titer. She had no history of systemic disease or co-morbidity, nor was she taking any medications or illicit substances. The patient was treated with Rituximab (RTX) 1 g 2 weeks apart. Six months later, the CD19^+^ cells had not dropped below three cells/μl and nor had T_reg_ cells, which have been found to increase in membranous nephropathy (MN) patients responsive to RTX [1], showed an elevation. Nevertheless, serum PLA2R-antibodies turned negative. Despite the disappearance of anti-PLA2R antibodies, sCr increased to 2.9 mg/dl and proteinuria rose to 13 g/day after 9 months since RTX. The patient underwent a renal biopsy. Histology, examined on 36 glomeruli, revealed eight glomeruli with global and two with segmental sclerosis, without signs of interstitial involvement. The remaining 26 glomeruli showed the classical features of MN. At immunofluorescence, IgG4 (3+), C3 (+), C1q (±) deposition in the subepithelial spaces and positive staining for PLA2R (despite negative results in serum) were found. She received two doses of obinutuzumab (1000 mg, 2 weeks apart). The rationale of choosing obinutuzumab among available options was based on the detection of incomplete CD19 disappearance and failure of T_reg_ cells to increase combined with the presence of decompensated nephrotic syndrome. Follow-up data after the kidney biopsy are shown in Supplementary Table S2.

## COMMENT

Might a patient with such clinical presentation have something different from MN? Most likely not. The more recent KDIGO guidelines [2] support the legitimacy of a diagnosis, which should be histological by definition, by means of serological tools alone. Discussion on the need for biopsy-confirmed diagnosis for glomerulonephritis has been very fashionable in recent years [3–5].

The case of MN presented herein is undoubtedly unusual. Commonly, in patients with MN showing anti-PLA2R antibodies turning negative after RTX treatment, often after a single small dose [6], a subsequent decrease (and certainly not an increase) in proteinuria is expected. Moreover, renal function remains preserved at length unless decompensated nephrotic syndrome with AKI occurs, as did in our case. Serological data and clinical evolution might not always assure an optimal management of the patient in the absence of histological features. This case opens the discussion on the indication for kidney biopsy. Incidentally, in this case, while not critical from a therapeutic point view, the unusual proportion of sclerotic glomeruli was relatively surprising considering the patient's data at onset.

MN has traditionally been classified into two main forms: primary and secondary. The term “primary” referred to the lack of association with any identifiable systemic disease, infections, or drug exposure. MN was defined as “secondary” when combined with any systemic disease, including autoimmune conditions, infections, cancers, hematopoietic stem cell transplantation, sarcoidosis, and exposure to certain drugs or toxins. The discovery of the target antigen M-type PLA2R in 2009 [7] and the possibility to detect anti-PLA2R antibodies in serum paved the way to a revolutionary change of the classification of MN [8]. Serum anti-PLA2R autoantibody titers have been found to be highly specific diagnostic and prognostic biomarkers, and circumstantial evidence suggested that the antibody is pathogenic in most cases. Nowadays, 11 more antigens have been identified [9], overall accounting for ∼90% of membranous nephropathies. These antigens are likely relevant for diagnosing specific subsets of MN and immunohistochemistry/immunofluorescence determination of some antigens, i.e. Thrombospondin type-1 domain-containing 7° (THSD7A), Neural epidermal growth factor-like 1 protein, Protocadherin 7, and Exostosis 1/Exostosis 2, is becoming widespread. Incidentally, serological testing is currently available only for the detection antibodies to PLA2R and THSD7A [9].

Based on the distinct clinical and pathological features related to the different antigens, the Mayo Clinic Consensus Report proposes a two-step classification: (i) identification of the target antigen, whenever possible, followed by (ii) the search for an underlying systemic disease or associated condition. In this Consensus, the need for renal biopsy to diagnose MN in patients with positive serology for anti-PLA2R antibodies was also addressed [8].

### Is serology a sufficient tool to manage patients with membranous nephropathy?

A non-invasive approach to the diagnosis might seem to be appealing for first-level nephrological units with scarce attitude to renal biopsy and little experience with this procedure. However, these units also have restricted laboratory facilities (rendering the accuracy of detection of antibodies to target antigens, including anti-PLA2R antibodies, questionable, especially in immunofluorescence). Therefore, these units probably have limited experience in following these patients on serological grounds (especially when performed in both immunofluorescence (IF) and solid-phase assays, as suggested by international guidelines).

On the other hand, performing a renal biopsy at an experienced center is nether time-consuming nor disturbing for the patient, especially when conducted on an outpatient basis. Aside from these considerations, with the important exception of patients with PLA2R antibodies, absence of secondary causes, no diabetes, and eGFR >60 ml/min, renal biopsy might still assist in guiding treatment decisions and provide valuable prognostic insights.

Nevertheless, it is worth mentioning that one-fits-all approach might not work in this setting: while a renal biopsy may not be necessary from a strictly diagnostic perspective at the first observation of patients with the characteristics mentioned above, it might become desirable later if the patient is unresponsive to therapy or experiences worsening kidney function.

In 2019, Bobart *et al.* [10], reported the Mayo experience, showing that a positive test for anti-PLA2R serology in patients who presented with nephrotic syndrome, normal kidney function, and no evidence of another process to account for proteinuria was sufficient to make a diagnosis of primary MN without the need for a diagnostic kidney biopsy. This has been widely accepted in the nephrology community [11, 12], and has been added to the KDIGO 2021 guidelines [2]. However, PLA2R serology in the Mayo Clinic cohort was based both on enzyme-linked immunosorbent assay plus IF and one should acknowledge that the laboratory expertise and availability for conducting both IF and solid-phase assays for anti-PLA2R are not consistent globally.

Additionally, limited information exists regarding the timing of biopsy as related to anti-PLA2R Ab testing. Timing is crucial, as anti-PLA2R Ab titers vary not only based on disease progression but also as a consequence of therapy, especially immunosuppression, and immunological remission status [12].

From a therapeutic perspective, the efficacy of Rituximab (RTX) has been proven when used as a first-line treatment of standard cases of MN, i.e. young adults presenting with nephrotic syndrome and normal renal function, having positive anti-PLA2R antibodies [13]. However, RTX is not the only therapeutic option, especially in patients with heavy proteinuria and high levels of anti-PLA2R antibodies [2]. Indeed, while low titers are associated with a higher probability of remission, high levels of anti-PLA2R antibodies are related to a higher risk of disease relapse and a lower probability of remission following treatment [14, 15]. Referring to our case, when the patient had normal serum creatinine levels, an initial course of Rituximab was administered. The subsequent development of definite renal impairment, which was not due to acute tubular necrosis (since the tubules were found to be perfectly normal) but to the effects of decompensated nephrotic syndrome, along with the failure to respond to Rituximab were the main reasons for proceeding with a renal biopsy. Based on the histologic features and the flowcytometry profile (incomplete disappearance of CD19^+^ cells and absence of T_reg_ increase) [1], a stronger B-cell depleting agent instead of exploring other therapeutic options has been chosen.

### Are patients with MN homogeneous in real life?

We have recently [unpublished] performed a cluster analysis in a large cohort of 108 patients with MN. The analysis revealed the presence of three separate clusters showing distinct differences in kidney function and histopathological characteristics. In brief, Cluster 1 exhibited the highest levels of tubular atrophy and interstitial fibrosis, with the lowest estimated glomerular filtration rate (eGFR) at both baseline and 12 months. Cluster 2 showed moderate levels of fibrosis and sclerosis, with intermediate eGFR values, while Cluster 3, characterized by the lowest levels of fibrosis and sclerosis, had the highest eGFR at both time points. Additionally, Cluster 1 demonstrated the highest levels of PLA2R-antibodies after 12 months, suggesting a possible association with worse outcomes compared to the other clusters.

In our cohort, patients with GFR > 60 ml/min were 49 of 108 (45.4%) (Table 1). Of these patients, 28 had PLA2R-antibodies in serum (57% of patients with GFR > 60 ml/min, but 26% of the entire cohort). This was the subgroup that met the requirements of GFR > 60 ml/min, positive PLA2R-antibodies, absence of comorbidities for whom renal biopsy may not add useful elements to management. Four out of 49 similar patients with GFR > 60% (8%) were negative in serum and positive by tissue histochemistry. These patients would not have been identified without renal biopsy. The same applies to the other 17 of 49 patients (34.7%) with GFR > 60% who were PLA2R-antibody-negative in both serum and tissue. Therefore, the patients with putative MN who do not need a diagnostic renal biopsy (i.e. those with GFR >60 ml/min, positive PLA2R-antibody, absence of comorbidities) represent a discrete subset, but not the majority [unpublished]. Consonant with our observation, in a retrospective Chinese cohort study of 262 patients with PLA2R-associated MN, total renal chronicity score calculated according to the degree of glomerulosclerosis, interstitial fibrosis, tubular atrophy, and arteriosclerosis was applied to evaluate renal chronicity. During a median follow-up of 24.5 months, both a higher total renal chronicity score and non-minimal chronicity were found to be independent risk factors for renal outcome [16]. When interpreting our data, however, one cannot exclude the possibility of a center effect in our cohort, as our center serves as a referral hub for rare and complex cases in Northwest Italy.

**Table 1:** Main characteristics of the 49 patients with GFR >60 ml/min at baseline.

	0 baseline	12 months
Age (year)	59.35 [16–85]	
sCr (mg/dl)	0.88 [0,5–1,37]	0.85 [0,5–2,5]
eGFR ml/min	87.7 [60,1–134]	90.65 [23–120]
Proteinuria (g/24 h)	4.9 [3,7–12,8]	0.89 [0,1–7,1]

Data are expressed as median, [min–max] unless different specified.

### Kidney biopsy for whom?

Our previous research demonstrated that kidney biopsies can achieve an optimal safety profile, even when performed as an outpatient procedure in selected referral centers [17, 18].

The paradigm of the importance of performing kidney biopsy should be also contextualized in the general scenario of autoimmune conditions with renal involvement. This could also be the case for lupus nephritis (and indeed, rheumatologists often dismiss the problem and embark on immunosuppressive treatments without renal biopsy) or antineutrophil cytoplasmic antibody-associated vasculitis (again with evident deviations from diagnostic appropriateness by rheumatologists, immunologists, and, disappointingly, sometimes also nephrologists). Not to mention cryoglobulinemic nephritis, which often represents a blasphemous path of managing patients without histological insights when handled by internists, infectious disease specialists, rheumatologists, immunologists, and, again, sometimes even nephrologists. A recent survey on 1515 patients presumed to have IgA nephropathy recruited all over the world (Europe, UK, USA, China, and Japan) revealed that one-fifth of patients received a diagnosis based on clinical grounds [19] (Kidney Week 2022). Luckily, renal biopsy has been stated to be mandatory prior to treatment in patients with lupus nephritis [3] and in patients with putative renal involvement by cryoglobulinemia [4]. Renal biopsy is crucial in patients with antineutrophil cytoplasmic antibody-associated vasculitis as well, albeit it can be postponed to initial therapy in the presence of life-threatening clinical conditions [5]. The etiquette we were taught as children prevents us from commenting on the practice of diagnosing mesangial nephropathy with dominant IgA deposits without renal biopsy.

It is important to recognize that there may be heterogeneity in terms of safety, accessibility, feasibility, and costs when considering the procedural aspects of performing a kidney biopsy. Our previous research demonstrated that kidney biopsies can achieve an optimal safety profile in selected referral centers, even in high-risk patients (e.g. under dialysis), and when performed as an outpatient procedure [17]. This is true also for systemic diseases with kidney involvement [18], and is especially true for *a priori* primary MN. Indeed, in our cohort of 108 cases we observed 0.9% minor complications (never requiring hospital stay), and no major complications [14].

We are aware that traditional histopathology only scratches the surface of what information can be obtained from kidney tissue. The use of microdissection and “omic” techniques have the potential to improve our understanding of renal injury beyond histopathology. When combined with clinical data, ongoing research is exploring the role of molecular insights from kidney biopsies aiming to optimize existing therapies and identify further therapeutic targets [16]. Nevertheless, due to costs and availability limits, the implementation of these techniques might remain a prerogative of very few centers. However, when a kidney biopsy is performed for research beyond diagnostic/prognostic purposes, approval from the competent IRB and informed consent are required.

We had the honor to be invited by the organizers to participate in the Mayo Clinic Consensus Meeting held in October 2022 in Rochester. As regard to the delicate point of the indications to renal biopsy in MN, and coming back to our clinical case, herewith the view of the G. Bosco Hub Hospital in Turin: in patients who present with nephrotic syndrome and normal kidney function with positive anti-PLA2R serology and no evidence of another process to account for proteinuria renal biopsy could be postponed for at least 12 months taking the time to verify the effect of a complete RTX course. However, trends in proteinuria and anti-PLA2R levels need to be monitored, reserving kidney biopsy to cases with no significant improvement. This observation stemmed from our experience, showing persistent proteinuria after 12 months from RTX in a consistent number of patients despite the absence anti-PLA2R at 1-year follow-up, especially if T_reg_s fail to increase [1].

In conclusion, while we are convinced that the path forged by the Mayo Clinic investigators is the one that will prevail in the near future, we believe that in a not negligible number of patients renal biopsy might remain a non-renounceable tool for the management of MN in real life. With the important exception of the specific subset of patients who are anti-PLA2R positive, have an eGFR >60 ml/min, no secondary cause of MN, and no diabetes—who likely do not benefit from a renal biopsy for therapeutic strategy—in the other categories, the need for a renal biopsy, despite a clinically suggestive diagnosis, is supported by the significant additional information that can be obtained from the histological findings.

## Supplementary Material

sfae292_Supplemental_File

## Data Availability

Not applicable.
