# Supplementary material for: The role of kidney biopsy in the diagnosis of membranous nephropathy
Source: Clin Kidney J. 2024 Oct 3;17(10):sfae292. doi: 10.1093/ckj/sfae292 (PMC11491826; doi:10.1093/ckj/sfae292)
Supplement: sfae292_Supplemental_File [file sfae292_supplemental_file.docx]

***Table 1s: Baseline investigations***

| **Baseline investigations** |  |
| --- | --- |
| sCr (md/dL) | 1.1 |
| Proteinuria (g/24h) | 7 |
| anti-PLA-2R | 97 RU/ml  IF+++ |
| C3 (mg/dL) | 1.72 |
| C4 (mg/dL) | 0.26 |
| Anti-dsDNA | Negative |
| ANA | Negative |
| ENA | Negative |
| Hepatitis C | Negative for anti-HCV antibodies |
| Hepatitis B | anti-HBs+; anti-HBc neg. HBsAg negative. |

***Table 2S: investigations during follow-up after kidney biopsy.***

|  | **At the time of the biopsy**  **(before Obinutuzumab)** | **3 months after biopsy** |
| --- | --- | --- |
| sCr (mg/dle) | 2,9 | 1.4 |
| Proteinuria (g/24h) | 13 | 8,3 |
| Albumin (g/l) | 26 | 38 |
| serum anti-PLA-2R RU/ml | Negative  (<3RU and negative PLA2R in IF)  positive staining for PLA2R | Negative  (<3RU and negative PLA2R in IF) |
